# XRCC5 cooperates with p300 to promote cyclooxygenase-2 expression and tumor growth in colon cancers

**DOI:** 10.1371/journal.pone.0186900

**Published:** 2017-10-19

**Authors:** Zhifeng Zhang, Fufu Zheng, Zhenlong Yu, Jiajiao Hao, Miao Chen, Wendan Yu, Wei Guo, Yiming Chen, Wenlin Huang, Zhijun Duan, Wuguo Deng

**Affiliations:** 1 The First Affiliated Hospital & Institute of Cancer Stem Cell, Dalian Medical University, Dalian, China; 2 The First Affiliated Hospital, Sun Yat-sen University, Guangzhou, China; 3 SunYat-sen University Cancer Center, State Key Laboratory of Oncology in South China, Collaborative Innovation Center of Cancer Medicine, Guangzhou, China; 4 State Key Laboratory of Targeted Drug for Tumors of Guangdong Province, Guangzhou Double Bioproduct Inc., Guangzhou, China; University of South Alabama Mitchell Cancer Institute, UNITED STATES

## Abstract

Cyclooxygenase (COX) is the rate-limiting enzyme in prostaglandins (PGs) biosynthesis. Previous studies indicate that COX-2, one of the isoforms of COX, is highly expressed in colon cancers and plays a key role in colon cancer carcinogenesis. Thus, searching for novel transcription factors regulating COX-2 expression will facilitate drug development for colon cancer. In this study, we identified XRCC5 as a binding protein of the COX-2 gene promoter in colon cancer cells with streptavidin-agarose pulldown assay and mass spectrometry analysis, and found that XRCC5 promoted colon cancer growth through modulation of COX-2 signaling. Knockdown of XRCC5 by siRNAs inhibited the growth of colon cancer cells *in vitro* and of tumor xenografts in a mouse model *in vivo* by suppressing COX-2 promoter activity and COX-2 protein expression. Conversely, overexpression of XRCC5 promoted the growth of colon cancer cells by activating COX-2 promoter and increasing COX-2 protein expression. Moreover, the role of p300 (a transcription co-activator) in acetylating XRCC5 to co-regulate COX-2 expression was also evaluated. Immunofluorescence assay and confocal microscopy showed that XRCC5 and p300 proteins were co-located in the nucleus of colon cancer cells. Co-immunoprecipitation assay also proved the interaction between XRCC5 and p300 in nuclear proteins of colon cancer cells. Cell viability assay indicated that the overexpression of wild-type p300, but not its histone acetyltransferase (HAT) domain deletion mutant, increased XRCC5 acetylation, thereby up-regulated COX-2 expression and promoted the growth of colon cancer cells. In contrast, suppression of p300 by a p300 HAT-specific inhibitor (C646) inhibited colon cancer cell growth by suppressing COX-2 expression. Taken together, our results demonstrated that XRCC5 promoted colon cancer growth by cooperating with p300 to regulate COX-2 expression, and suggested that the XRCC5/p300/COX-2 signaling pathway was a potential target in the treatment of colon cancers.

## Introduction

Colon and rectal cancer (colorectal cancer, CRC) is the third most common carcinoma, and has become one of the leading causes of death from cancers worldwide [1]. In the latest cancer statistics published in 2017, the American Cancer Society estimates that CRC alone accounts for 9% of all new cancer cases in males and 8% of all new cancer cases in females in the United States [2]. Moreover, 9% of all cancer related death in males and 8% of all cancer related death in females can be attributed to CRC [2].Major advances in the understanding of CRC biology have led to the development of new diagnostic and prognostic biomarkers, and the development of novel molecular targeted therapies for CRC. However, improvement of the five-year survival rates of CRC patients still mainly relies on diagnosis at early stages, and only curative surgical resection has the possibility to cure early staged CRC. When CRC develops into advanced stages, curative surgical resection is nearly impossible. To date, the combination therapy with cytotoxic drugs including 5-fluorouracil, leucovorin, oxaliplatin, and capecitabine is the first-line chemotherapy for advanced CRC with metastasis [1, 3, 4]. However, the efficacy of the combination therapy with cytotoxic drugs for advanced staged CRC remains limited due to a combination of drug toxicity and resistance. With intensive studies on the molecular mechanisms in CRC development, novel treatment targets for CRC are identified. Bevacizumab (a vascular endothelial growth factor A antibody) and cetuximab (an epidermal growth factor receptor antibody) have been proved effective to treat advanced CRC with clinical trials [1, 4]. However, because of multiple signaling pathways involved in CRC carcinogenesis and development, when one pathway is inhibited, other compensatory pathways could be activated. So it is not uncommon that CRC patients can also develop drug resistance to bevacizumab and cetuximab. Thus searching for novel therapeutic targets for advanced CRC to maximize survival time is of great significance to both patients and clinicians.

Cyclooxygenase (COX) is the rate-limiting enzyme in prostaglandins (PGs) biosynthesis. In mammals, COX catalyzes the conversion of arachidonic acid to prostaglandin G2 (PGG2), PGG2 is then converted to prostaglandin H2 (PGH2), which is ultimately converted to various prostanoids by specific prostanoid synthases [5–7].There are two major isoforms of COX named with COX-1 and COX-2, and their expression patterns and associations with terminal prostanoid synthases are distinct. COX-1 is expressed constitutively in most normal tissues, and associates with cytosolic PGE synthases [8]. Correspondingly, COX-2 is induced to express in response to hormones, cytokines, and growth factors, and associates with membrane-bound PGE synthases [7, 9]. Accumulating evidence has indicated that COX-2 plays key roles in carcinogenesis and cancer progression. PGE2 as a product of COX-2 introduces extracellular signals into target cells via G protein coupled receptor (GPCR) family on cellular membrane [10]. After coupled with GPCR, PGE2 activates Ras and phosphatidylinositol 3-kinase (PI3K) pathways to inhibit apoptosis of colon cancer cells [11]. PGE2 can also activate Ras-mitogen-activated protein kinase signaling cascade to promote intestinal adenoma proliferation [12]. Moreover, COX-2 increases the expression of vascular endothelial growth factor (VEGF) of colon cancer cells, which has the ability to promote angiogenesis of tumors [13]. Furthermore, COX-2 activates the NOTCH and WNT signaling pathways to promote cancer stem cell formation [14]. The immunological characteristic of tumor microenvironment is the shift from a Th1 predominant immune response to a Th2 predominant one [15]. Tumor necrosis factor-α (TNF-α), interferon-γ (IFN-γ), and interleukin-2 (IL-2) increase Th1 cell proliferation. Correspondingly, interleukin-4 (IL-4), interleukin-6 (IL-6), and interleukin-10 (IL-10) promote Th2 cell proliferation. Previous studies have shown that COX-2 decreases the expression of TNF-α, IFN-γ and IL-2, and increases the expression of IL-4, IL-6 and IL-10 in tumor cells. Thus COX-2 can facilitate immune response shift in tumor microenvironment, which facilitates tumor cells to evade from host immune surveillance [15–17]. In summary, COX-2 promotes carcinogenesis and cancer progression through participating in promoting cell proliferation, inhibiting cell apoptosis, enhancing angiogenesis, promoting cancer stem cell formation, and facilitating immune response shift in tumor microenvironment, COX-2 is deemed to be a promising molecular target for CRC treatment. One randomized controlled trial has shown that COX-2 inhibitor celecoxib prevents the occurrence of colonic adenoma, which is a precancerous condition of CRC [18]. A meta-analysis also shows that aspirin (a nonselective COX inhibitor) improves CRC patients survival especially in CRC patients with high COX-2 expression in tumor issues [19]. These clinical data further support COX-2 as a promising target for CRC treatment. However, the preventive and treatment effects of available COX-2 inhibitors (aspirin and celecoxib) on CRC are not ideal, and most CRC patients failed to respond to the available COX-2 inhibitors. It is known that the expression of COX-2 is regulated at transcriptional and translational levels, therefore it is of great significance to find novel factors acting at the above two levels to regulate COX-2 expression, which would facilitate new therapeutic development for CRC.

X-ray repair cross-complementing protein 5 (XRCC5) also called Ku80 is encoded by XRCC5 gene located in 2q33-35 [20]. XRCC5 and XRCC6 form an XRCC5/XRCC6 heterodimer that is a DNA-dependent protein kinase complex (DNA-PK) [20, 21]. XRCC5/XRCC6 heterodimer binds the ends of broken DNA double strands to accomplish DNA non-homologous end joining repair to maintain stability of the whole genome and chromosomes [22]. Studies also indicate that the function of XRCC5 is not limited to DNA double strand breaks repairs, XRCC5 can also act as an adherence factor participating in cellular adherence, migration, and invasion of tumors [23, 24]. Additionally, XRCC5 has been proved to be overexpressed in various tumor tissues (including CRC), which implies that XRCC5 is a tumor promoting factor [25–28]. However, little is known about the molecular mechanisms of XRCC5 participating in CRC carcinogenesis and development. As XRCC5 has the ability to bind DNA strands that is also a characteristic of transcription factors, we hypothesize that XRCC5 may act as a transcription factor to promote oncoprotein expression and participate in CRC development. Our previous study has shown that XRCC5 binds to COX-2 gene promoter and increases COX-2 expression to promote lung cancer cell proliferation [29]. Whether the same interaction between XRCC5 and COX-2 gene promoter also exists in CRC is not clear. Moreover, p300 (a transcription co-activator) has been reported to participate in the transcription of COX-2 [30], whether p300 participates in the interaction between XRCC5 and COX-2 gene promoter in CRC also needs to be elucidated.

In this study, we identified XRCC5 as a binding protein of the COX-2 gene promoter in colon cancer cells with streptavidin-agarose pulldown assay and mass spectrometry analysis, and found that XRCC5 promoted colon cancer growth through modulation of COX-2 signaling. Knockdown of XRCC5 by siRNAs inhibited the growth of colon cancer cells *in vitro* and of tumor xenografts in a mouse model *in vivo* by suppressing COX-2 promoter activity and COX-2 protein expression. Conversely, overexpression of XRCC5 promoted growth of colon cancer cells by activating COX-2 promoter and increasing COX-2 protein expression. Moreover, the role of p300 acetylating XRCC5 to co-regulate COX-2 expression was also evaluated. Our study suggests that the XRCC5/p300/COX-2 signaling pathway is a potential target in the treatment of colon cancers.

## Material and methods

### Cell lines and culture conditions

Human colon cancer cell lines (SW480, LoVo, DLD-1, RKO) were obtained from American Type Culture Collection (ATCC, Manassas, VA) and cultured in RPMI 1640 media (Gibco) supplemented with 10% heat-inactivated fetal bovine serum(Biological Industries), 100mg/ml penicillin and 100mg/ml streptomycin (Hyclone), and maintained in an incubator with a humidified atmosphere of 95% air and 5% CO_2_ at 37°C.

### Streptavidin-agarose pulldown assay

The biotin-labeled double-stranded oligonucleotide probe corresponding to -30/-508 fragments of COX-2 promoter sequence were synthesized by TAKARA Company. Primers of the oligonucleotide probe were as following: sense, 5’-ACGTGACTTCCT CGACCCTC-3’; antisense, 5’-AAGACTGAAAACCAAGCCCA-3’. Streptavidin- agarose pulldown assay was performed as following: 1) 400μg nuclear proteins from cell lines, 4μg of the double-strand biotin-labeled probe and 50μl of steptavidin-agarose beads solution (Sigma) were mixed and incubated on a rotating wheel at room temperature for 2 hours. 2) Steptavidin-agarose beads were pelleted by centrifugation at 600×g for 1min and washed with 200μl phosphate buffer saline (PBS) with protease inhibitors for three time. 3) Steptavidin-agarose beads were collected and resuspended with 30μl loading buffer, and then cooked at 100°C for 5min. 4) The supernatant containing the bound proteins was collected and then separated by 10%SDS-PAGE for further silver staining and mass spectrometry analysis.

### Identification of COX-2 promoter-binding proteins

The supernatant containing COX-2 promoter-binding proteins were separated by 10% SDS-PAGE. Silver staining was used to visualize protein bands according to the manufacturer's protocols (Beyotime, China). Then the protein bands of interest in the silver stained gel were cut, decolorized and digested with trypsin. Mass spectrometry analysis was utilized to identify the digested samples. Mass spectrometry data were then compared with those of the available proteomics databases to identify the proteins in the bands of interest.

### Plasmid vector, small interfering RNA (siRNA) and p300 HAT-specific inhibitor

A 5’-flanking DNA fragment from position -891 to +9 of human COX-2 gene was constructed into a promoter luciferase expression vector, pGL3. XRCC5 overexpression vector (XRCC5), wild type p300 overexpression vector (p300WT), p300 histone acetyltransferase (HAT) domain deletion mutantvector (Δp300), FLAG-p300 and the negative control vector FLAG-LacZ were purchased from Addgene. siRNAs targeting XRCC5 and negative controlsiRNAs were purchased from ShangHai GenePharma Company (Shanghai, China) (Si1: sense, 5’-GGCUCCAAUUUGUCUAUAATT-3’; antisense, 5’-UUAUAGACAAAUUGGA GCCTT-3’. Si2: sense, 5’-GGUGGCCAUAGUUCGAUAUTT-3’; antisense, 5’-A UAUCGAACUAUGGCCACCTT-3’. Si3: sense, 5’-GAGCAGCGCUUUAACAACU TT-3’; antisense, 5’- AGUUGUUAAAGCGCUGCUCTT-3’. Negative control (Sictr): sense, 5’-UUCUCCGAACGUGUCACGUTT-3’; antisense, 5’-ACGUGACACGUUCGGAGAATT-3’). A p300 HAT-specific inhibitor (C646) was purchased from AdooQ.

### Transfection of colon cancer cells

Colon cancer cells were transfected with XRCC5 overexpression vector, siRNAs of XRCC5 or negative control siRNAs (2μg) mediated by Lipofectamine 2000 (Invitrogen) as well as p300WT, Δp300, FLAG-p300, FLAG-LacZ and COX-2 promoter luciferase plasmids encapsulated with DC-nanoparticles in 6-well plates (2×10^4^ cells per well). After treatment with plasmid vectors and siRNAs for 48 hours, cells were harvested for further assays.

### Nuclear protein extraction and Western blot

Nuclear proteins of colon cancer cells or tumor tissues were collected in accordance with the protocols established in our previous article [29]. The concentration of the nuclear proteins was determined by BCA protein assay. 10% SDS-PAGE was used to separate nuclear proteins. Nuclear proteins were subsequently transferred to a polyvinylidene difluoride (PVDF) membrane. After protein transfer to PVDF membrane, PVDF membrane was blocked with 5% skim milk in Tris-buffered saline containing 0.05% Tween-20 (TTBS) and incubated with primary antibodies against XRCC5 (Abcam,1:500 dilution), COX-2 (Millipore, 1:500 dilution), p300(CST, 1:500 dilution), β-actin (Proteintech, 1:1000 dilution) and GAPDH (Proteintech, 1:1000 dilution) overnight at 4°C. PVDF membrane was subsequently incubated with the secondary antibody at 37°C for 2 hours. The band was photographed and quantified with enhanced chemiluminescence system. The intensity of β-actin or GAPDH was used as the internal reference.

### Reverse transcription-polymerase chain reaction (RT-PCR)

Colon cancer cell lines with according treatments were cultured for 48 hours. Total RNA of colon cancer cells was extracted with Trizol Reagent (TaKaRa) according to the manufacturer’s instructions. The PCR primers corresponding to COX-2, XRCC5 and GAPDH functional gene sequences were synthesized by TaKaRa. The primer sequences were as following: 1) COX-2: sense, 5’-TCACAGGCTTCCATTGACCAG-3’; antisense, 5’-CCGAGGCTTTTCTA CCAGA-3’. 2) XRCC5: sense, 5’-TGACTTCCTGGATGCACTAATCGT-3’; antisense, 5’-TTGGAGCCAATGGTCAGTCG-3’. 3) GAPDH: sense, 5’–AATCC CATCACCATCTTCC-3’;antisense, 5’-CATCACGCCACAGTTTCC-3’. Optical density of the products at 260nm (A260 = 1 for 40μg/mL RNA) were measured to quantify RNA, and the ratio of the optical density obtained at 260 and 280 nm (pure RNA: A260/A280 = 2.0) was calculated to determine the purity of RNA. All the optical analyses were carried out with the UV-1206 spectrophotometer (Shimadzu). Reverse transcription PCR was performed with a RNA PCR Kit (Takara) according to the manufacturer’s instructions. The samples were denatured at 98°Cfor 3min, and followed by 30 PCR cycles (10s at 98°Cfordenaturation, 30s at 58°C for annealing, and 30s at 72°C for elongation). PCR products were then separated by 1.5% agarose gel electrophoresis. Bands were visualized in ultraviolet light and photographed subsequently. The intensity of GAPDH was used as the internal reference.

### Luciferase reporter assay

LoVo cells or RKO cells (200,000 cells per well) plated in six-well plates were transfected with the COX-2 promoter luciferase plasmids encapsulated with DC-nanoparticles. LoVo cells were subsequently co-transfected with siRNAs of XRCC5 (Si1, Si2 and Si3) (2μg) or negative control siRNAs (2μg) mediated by Lipofectamine 2000 (Invitrogen) or treated with lipopolysaccharides (LPS) (10μg/ml). RKO cells were subsequently co-transfected with XRCC5 overexpression vector (2μg) and negative control FLAG-LacZ (2μg). And PBS was added to some wells of cells for negative control. 48 hours after treatment, the luciferase activity was measured using a luciferase reporter assay kit (BioVision) according to the protocols of manufacturer. Cells treated with PBS negative control was used for data alignment.

### Cell viability assay

LoVo or RKO cells were seeded in a 96-well culture plate at a density of 2×10^4^ cells per well, and cultured overnight. LoVo cells were co-transfected with siRNAs of XRCC5(Si1, Si2 and Si3) (2μg) or negative control siRNA (2μg) mediated by Lipofectamine 2000 (Invitrogen). RKO cells were co-transfected with XRCC5 overexpression vector (2μg), negative control FLAG-LacZ (2μg) or treated with celecoxib (25μM). LoVo cells were also treated with C646 (30uM) or transfected with p300WT vector (2μg), Δp300 vector (2μg), FLAG-p300 (2μg) or FLAG-LacZ (2μg). And PBS was added to some wells of cells for negative control. 48 hours after treatment, cell viability was evaluated using the MTS assay (CellTiter 96® AQueous One Solution Cell Proliferation Assay, Promega) according to the manufacturer’s instructions. The absorbance at 490 nm was recorded using a BioTek ELx800 absorbance microplate reader. Cells treated with PBS negative control or FLAG-LacZ negative control were used for data alignment.

### Clone formation assay and morphology inspection

LoVo cells (1,000 cells per well) seeded in six-well plateswere transfected with siRNAs of XRCC5(2μg) or negative control siRNAs (2μg)mediated by Lipofectamine 2000 (Invitrogen). After cultured for 14 days, colon cancer cells in six-well plates were washed with PBS, and subsequently fixed with fixation solution (methanol: glacial: acetic 1:1:8) for 10min. After fixation, six-well plates containing colon cancer cells were added with 0.1% crustal violet to stain for 30min. The clones only with more than 50 cells were counted and photographed under an optical microscope. Morphology of LoVo cells was also evaluated and photographed under an optical microscope.

### Immunofluorescence and confocal microscopy

Colon cancer cells were seeded onto coverslips in a six-well plate and fixed with 4% paraformaldehyde (w/v) for 30min. Coverslips with colon cancer cells were then washed with PBS for 15 min and permeabilized with 0.2% (w/v) Triton X-100 in PBS for 5min. PBS containing 1% bovine serum albumin (BSA) was used to block for 30 min. Coverslips were subsequently incubated with the primary antibodies against XRCC5 or p300 diluted in PBS containing 10% BSA overnight. Coverslips were washed with PBS for 3 times and then incubated with secondary fluorescein isothiocyanate conjugated antibody or tetra methyl rhodamine isothiocyanate conjugated antibody for 1 hour. After washed with PBS for 3 times, coverslips with colon cancer cells were stained with DAPI (Beyotime). Leica DM 14000B confocal microscopy was employed to detect fluorescence and localize XRCC5 and p300 expressions in cells.

### Co-immunoprecipitation (co-IP) of p300/XRCC5 and acetylated XRCC5

Nuclear proteins were exacted from SW480, LoVo and RKO colon cancer cells. Nuclear proteins were incubated with a specific rabbit antibody against XRCC5 (Abcam) and a non immune rabbit IgG (Abcam) in each sample at 4°C overnight. Protein A/G agarose beads (Santa Cruz Biotechnology) were used to pull down the above immune complexes. Briefly, protein A/G agarose beads were added to the immune complexes and incubated at 4°C for 12 hours. Beads were then washed with ice-cold PBS containing protease inhibitors for 3 times. Loading buffers were added to the beads and boiled for 10min, and then centrifuged for 1min. Supernatant containing immunoprecipitated proteins of interest was subsequently separated by SDS-PAGE and p300 was detected by Western blot with a specific rabbit antibodyagainstp300 (CST). Correspondingly, a specific antibody against p300 (CST) was used to immunoprecipitate nuclear extract proteins, and Western blot with a specific antibody against XRCC5 (Abcam) was used to detect XRCC5 inimmunoprecipitated proteins. To detect acetylation of XRCC5 in nuclear proteins, a pan-Acetyl antibody (CST) for acetylated proteins was used to immunoprecipitate nuclear proteins, and Western blot with a specific antibody against XRCC5 (Abcam) was employed to detect XRCC5 in immunoprecipitated proteins.

### Immunoprecipitation of p300 domain

To further identify the specific domain in p300 for its interaction with XRCC5, we designed p300 overexpression vectors with its five different domains fused to flag tags (data not shown). LoVo cells were then transfected with p300 overexpression vectors with flag tags and cultured for 48 hours. Nuclear extracts of LoVo cells were then immunoprecipitated with anti-flag tag antibody (CST), and the precipitated complexes were then blotted with an antibody against XRCC5 (Abcam).

### Xenograft mouse model and tumor tissue processing

Nude mice were obtained from the SPF Laboratory Animal Center at Dalian Medical University. Armpits of nude mice were injected with LoVo cells (5×10^6^) subcutaneously. Once tumors were palpable, we measured tumor volumeevery two days with calipers. When tumor grew to a volume of 150mm^3^, nude mice were then divided into 4 groups (5 mice per group) randomly as following: a) siRNA of XRCC5treating group, b) negative control siRNA treating group, c) siRNA of XRCC5+LPS treating group and d) LPS treating group. In the negative control siRNA treating group, mice were injected intratumorally with 10μg negative control siRNAs conjugated with DC nanoparticles in 0.1 ml saline buffer twice a week for 17 days. In siRNA of XRCC5 treating group, mice were injected intratumorally with 10μg siRNA of XRCC5 (Si3) conjugated with DC nanoparticles in 0.1 ml saline buffer twice a week for 17 days. In LPS treating group, mice were injected ntratumorally with LPS (Sigma) (10μg/kg body weight) only once. In siRNA of XRCC5+LPS treating group, mice were treated with the combination of LPS and siRNA of XRCC5 (Si3) intratumorally. Tumor volume was calculated according to the equation of volume = (width^2^×length)/2. Seventeen days after the first treatment of each group, nude mice were sacrificed, and tumors were collected for further evaluation. Tumor size and weight of each mouse were measured and recorded. Fresh tumor tissues were mixed with lysis buffers or fixed in formalin for further analysis. Nuclear protein of tumor tissues was collected in accordance with the protocols established in our previous article [29]. Western blot was carried out to determine XRCC5 and COX-2 expression in tumors. Formalin-fixed tumor tissues wereparaffin-embedded and analyzed by immunohistochemistry for XRCC5 and COX-2 expression.

### Immunohistochemistry of tumor tissues in mice

Paraffin-embedded tumor tissues collected from mice were cut into slices with 4μm. Slices were then dewaxed with xylene and gradient alcohol. Tumor tissue slices were added with sodium citrate buffer (10mM) and microwaved for 4min to repair antigen. 3% H_2_O_2_ was added onto slices for 10min to block endogenous peroxidase. Slices were washed with PBS for three times (3min for one time).Tumor tissue slices were then added with primary antibodies against XRCC5 (Abcam) or against COX-2 (Millipore) with a dilution of 1:50 according to the manufacturer’s instructions. After washed with PBS for three times (3min for one time), slices were subsequently incubated with the secondary antibody at room temperature for 40min.Finally, DAB staining kit was used to visualize immunochemical staining.

### Statistical analysis

Statistical analysis was performed using SPSS16.0 statistical software package (SPSS Inc., Chicago, IL, USA). Independent Student’s t-test or one-way ANOVA analysis was employed to determine the difference of means among different groups. The values were presented as the mean±S.D. *p*<0.05 was considered statistically significant.

### Ethics statement

Animal experiments were carried out in accordance with animal care guidelines and protocols specifically approved by the Animal Experimental Ethical Committee of Dalian Medical University. All surgery was performed under anesthesia, and all efforts were made to minimize suffering.

## Results

### XRCC5 was identified as a COX-2 promoter-binding protein in colon cancer cells

**Fig 1:**
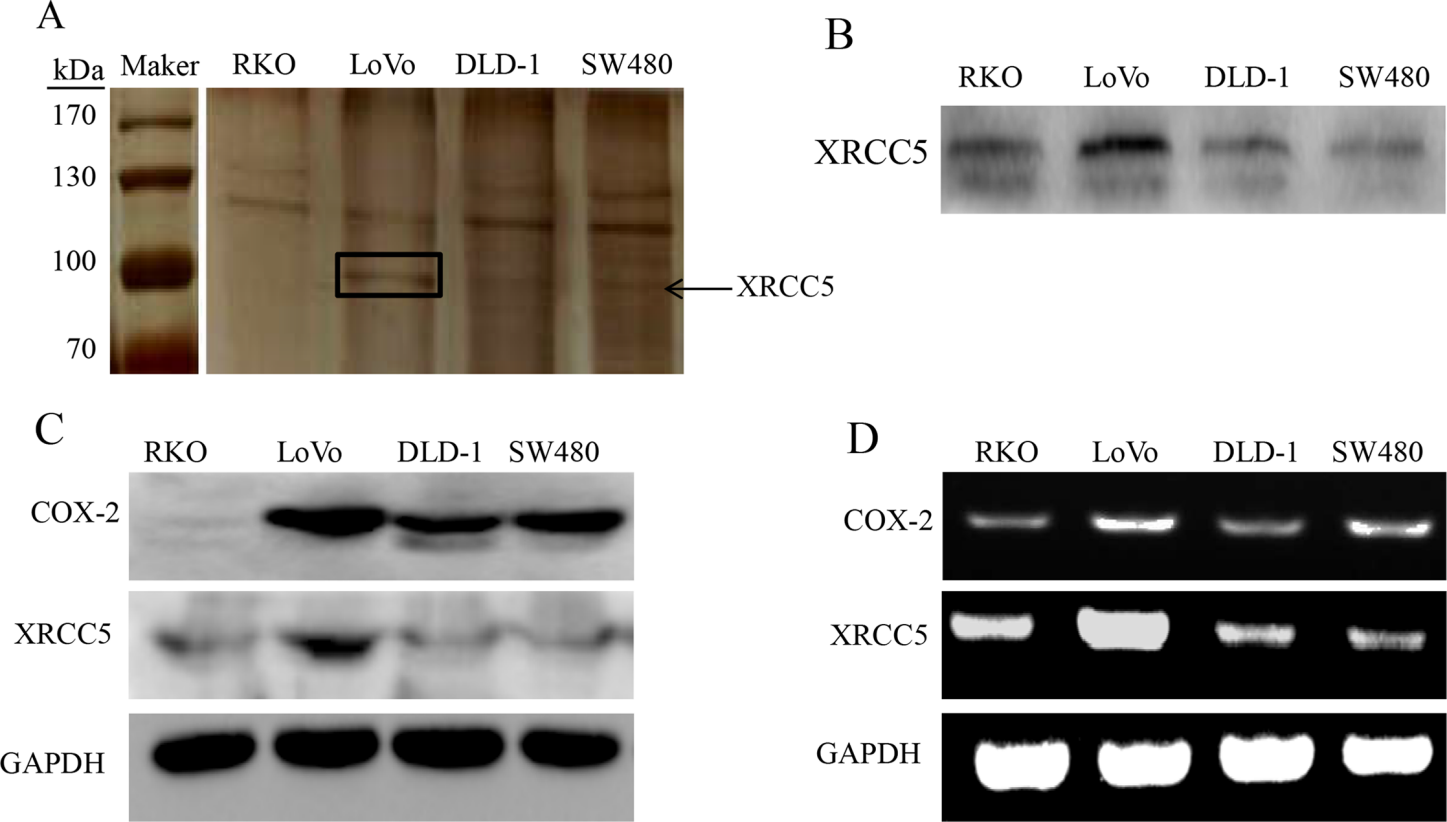
XRCC5 binding at the promoter region of COX-2 in colon cancer cells. (A) Streptavidin-biotin pulldown assay and SDS-PAGE with silver stain. A protein band with a molecular weight of 90-100kDa is indicated with an arrow in the figure. (B) Western blot with a specific antibody against XRCC5 in the pulled down proteins of RKO, LoVo, DLD-1 and SW480 cells. (C) Correlation between XRCC5 and COX-2 expression in protein level evaluated with Western blot in RKO, LoVo, DLD-1 and SW480 cells. (D) Correlation between XRCC5 and COX-2 expression in mRNA level evaluated with RT-PCR in RKO, LoVo, DLD-1 and SW480 cells.

A 479-bpbiotin-labeled double-stranded DNA probe corresponding to the 5’-flanking sequence of the COX-2 gene promoter region was synthesized according to our previous work [29]. Nuclear proteins extracted from human colon cancer cell lines (RKO, LoVo, DLD-1 and SW480) were incubated with biotin-labeled COX-2 promoter probes, and streptavidin-agarose beads were utilized to pull down nuclear proteins bound at COX-2 promoter region. As shown in Fig 1A, a protein band with a molecular weight of 90-100kDa was apparent in LoVo, DLD-1 and SW480 cells. Moreover, LoVo, DLD-1 and SW480 cells also had high COX-2 expression as shown in Fig 1C. However, the protein band with a molecular weight of 90-100kDa was not apparent in RKO cells, which also had low COX-2 expression as shown in Fig 1A and 1C. The identified protein band was cut and digested with trypsin. Mass spectrometry was then used to analyze the candidate protein. Mass spectrometric data of the protein was searched against an internationally recognized proteomics data library, and the protein was predicted to be XRCC5.To verify the results from Mass spectrometry, Western blot with a specific antibody against XRCC5 was utilized to analyze the nuclear proteins pulled down with biotin-labeled COX-2 promoter probes. As expected, XRCC5 was detected by Western blot with a specific antibody against XRCC5 in the pulldown samples, as shown in Fig 1B. To further evaluate whether a correlation between XRCC5 and COX-2 expression in colon cancer cells existed, we determined their expression in colon cancer cells (RKO, LoVo, DLD-1, and SW480) at protein and RNA levels respectively. Western blot and RT-PCR results indicated that the expressions of XRCC5 and COX-2 were positively correlated at both protein and mRNA levels, as shown in Fig 1C and 1D.The above results indicated that XRCC5 was a COX-2 promoter-binding protein in colon cancer cells.

### XRCC5 regulated COX-2 promoter activity and protein expression in colon cancer cells

**Fig 2:**
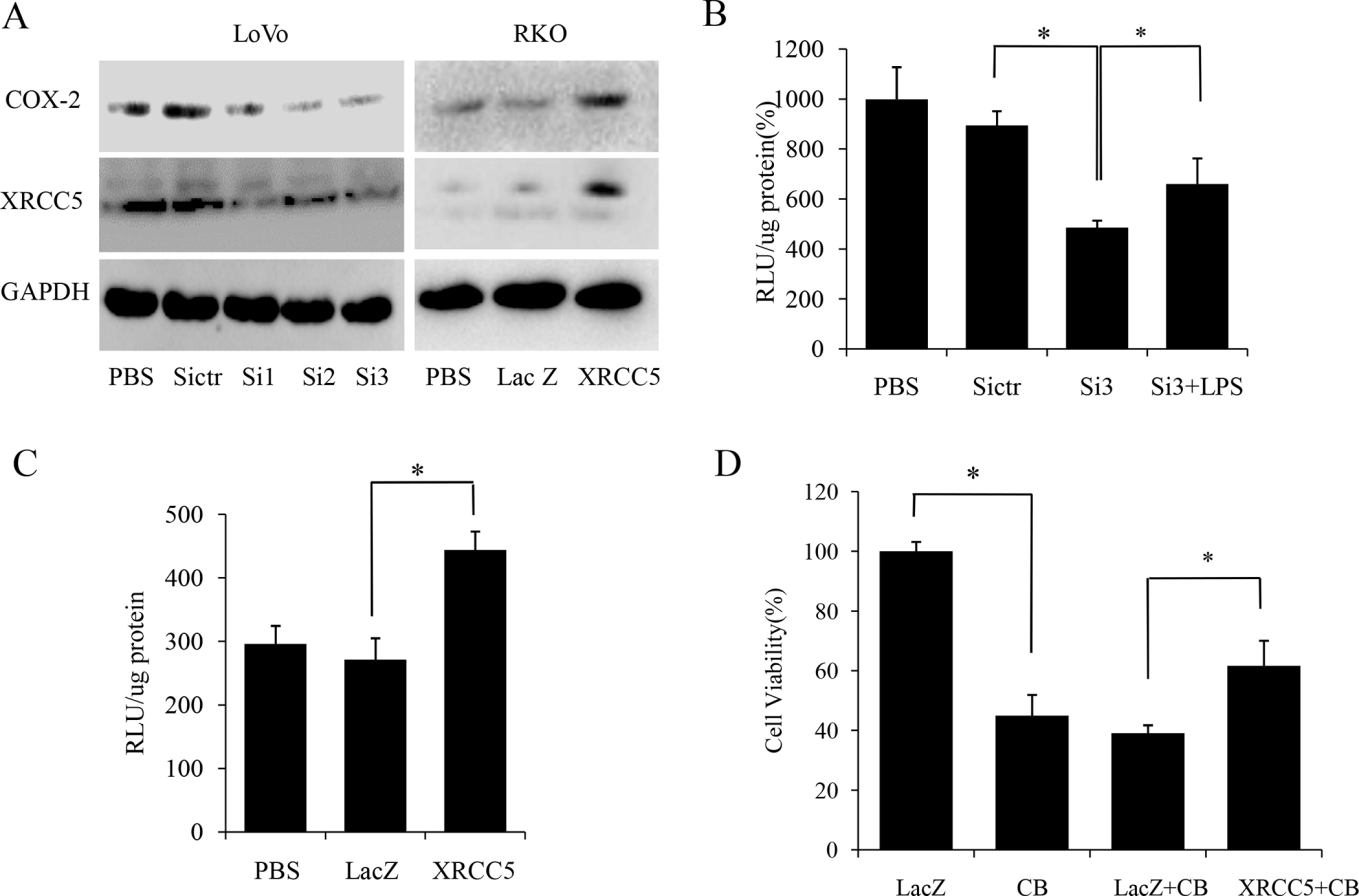
XRCC5 regulating COX-2 promoter activation and protein expression in colon cancer cells. (A) Left: Western blot of XRCC5 and COX-2 in LoVo cells. Right: Western blot of XRCC5 and COX-2 in RKO cells. (B) Luciferase reporter assay of the activity of COX-2 promoter in LoVo cells. Protein weight is used to adjust relative luciferase activity (RLU), and cells treated with BPS negative control are also used for data alignment. Data in the figure are presented as the meanmoter i*P*<0.05). (C) Luciferase reporter assay of the activity of COX-2 promoter in RKO cells. Protein weight is used to adjust relative luciferase activity (RLU). Data are presented as the meanve pro*P*<0.05).(D) MTS cell viability assay of RKO cells. Cells treated with LacZ is used for data alignment. Data are presented as the mean±SD. (*P<0.05). Si1, Si2 and Si3 represent three sequences of siRNAs of XRCC5, Sictr represents negative control siRNA of XRCC5, LacZ represents negative vector control, LPS represents lipopolysaccharides, PBS represents PBS negative control, XRCC5 represents overexpression of XRCC5, and CB represents celecoxib.

Streptavidin-agarose pulldown assay showed that XRCC5 bound to COX-2 promoter region. So XRCC5 might be a transcription factor of COX-2 and promote COX-2 expression in colon cancer cells. To verify this hypothesis, we designed three sequences of siRNA of XRCC5 (Si1, Si2 and Si3) to knock down XRCC5 expression to evaluate the effect of XRCC5 knockdown on COX-2 expression in LoVo cells. Western blot showed that all of the three sequences of siRNA suppressed the expression of COX-2, and the suppression effect of siRNA was marked with Si2 and Si3, as shown in Fig 2A. Conversely, we also evaluated the effect of overexpression of XRCC5 on COX-2 expression in RKO cells. RKO cells were transfected withXRCC5 overexpression plasmid to up-regulateXRCC5 levels, and the expression of COX-2was evaluated with Western blot. Western blot showed that overexpression of XRCC5 increased the expression of COX-2 as shown in Fig 2A. Next, we constructed luciferase-reporter vectors driven by COX-2 promoter. LoVo cells were transfected with luciferase-reporter vectors driven by COX-2 promoter, and then transfected with Si3 or treated with LPS. RKO cells were transfected with luciferase-reporter vectors driven by COX-2 promoter, and then transfected with XRCC5 overexpression plasmid. Luciferase reporter assay showed that knockdown of XRCC5 (Si3) decreased the activity of COX-2 promoter as compared with negative control siRNA of XRCC5 (Sictr) in LoVo cells, and LPS attenuated the suppression effect of XRCC5 knockdown on the activity of COX-2 promoter, as shown in Fig 2B. In contrast, overexpression of XRCC5 increased the activity of COX-2 promoter as compared with negative control (LacZ) in RKO cells as shown in Fig 2C. These results supported XRCC5 as a transcription factor regulating COX-2 promoter activity in colon cancer cells.

### XRCC5 promoted tumor cell proliferation in colon cancer cells

**Fig 3:**
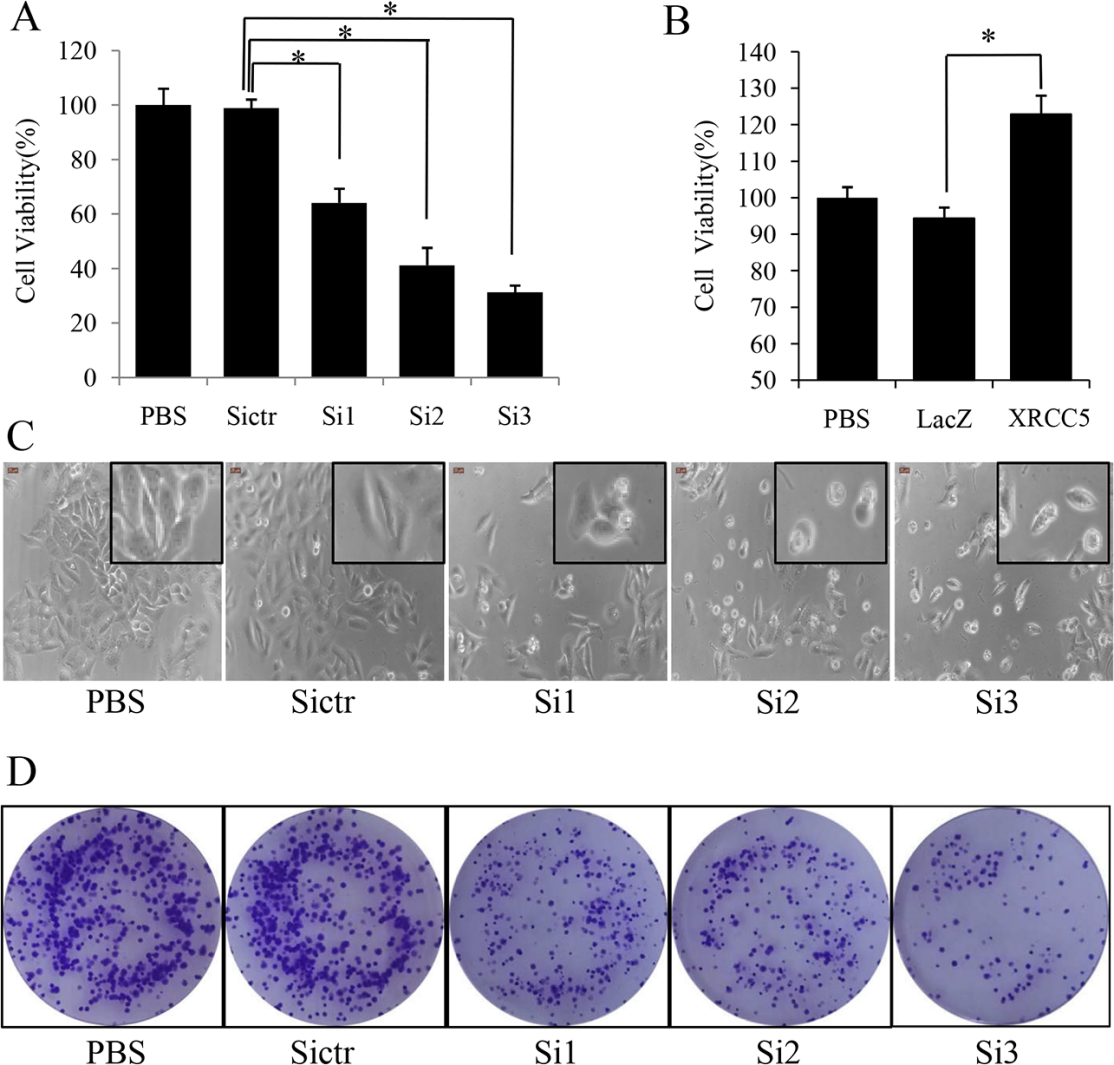
XRCC5 regulating colon cancer cell proliferation *in vitro*. (A) MTS cell viability assay of LoVo cells. Cells treated with BPS negative control are used for data alignment. Data are presented as the meaen.D. (*P<0.05). (B) MTS cell viability assay of RKO cells. Cells treated with PBS negative control are used for data alignment. Data are presented as the meannt.D. (*P<0.05). (C) Morphology observation of LoVo cells. (D) Colony formation assay of LoVo cells. Si1, Si2 and Si3 represent three sequences of siRNAs of XRCC5, Sictr represents negative control siRNA of XRCC5, PBS represents PBS negative control, XRCC5 represents overexpression of XRCC5, and LacZ represents negative control vector.

It is known that COX-2 plays a role in promoting cancer cell growth [11,12], and our results showed that XRCC5 bound to the promoter of COX-2 to up-regulate its expression. Therefore, we hypothesized that XRCC5 could promote colon cancer cell proliferation via increasing COX-2 expression. RKO cells were transfected with XRCC5 overexpression plasmid to up-regulate XRCC5 expression or treated with a COX-2 inhibitor celecoxib. LoVo cells were transfected with three sequences of siRNA of XRCC5 (Si1, Si2 and Si3) to knock down XRCC5 expression. MTS analysis was used to evaluate cell viability of RKO and LoVo cells. MTS showed that celecoxib decreased cell viability, and overexpression of XRCC5 attenuated the suppression effect of celecoxib on cell viability in RKO cells, as shown in Fig 2D. MTS also showed that all of the three sequences of siRNA decreased cell viability of LoVo cells, and the suppression effect of siRNA was marked with Si2 and Si3, as shown in Fig 3A. In contrast, overexpression of XRCC5 increased cell viability of RKO cells as shown in Fig 3B. Morphology observation also indicated low viability of LoVo cells with XRCC5 knockdown as shown in Fig 3C. Colony formation assay showed that XRCC5 knockdown decreased colony formation ability of LoVo cells as shown in Fig 3D. In summary, our data proved that XRCC5 promoted tumor cell proliferation via COX-2 in colon cancer cells.

### XRCC5 knockdown inhibited tumor growth by down-regulating COX-2 expression in a colon cancer mouse model

**Fig 4:**
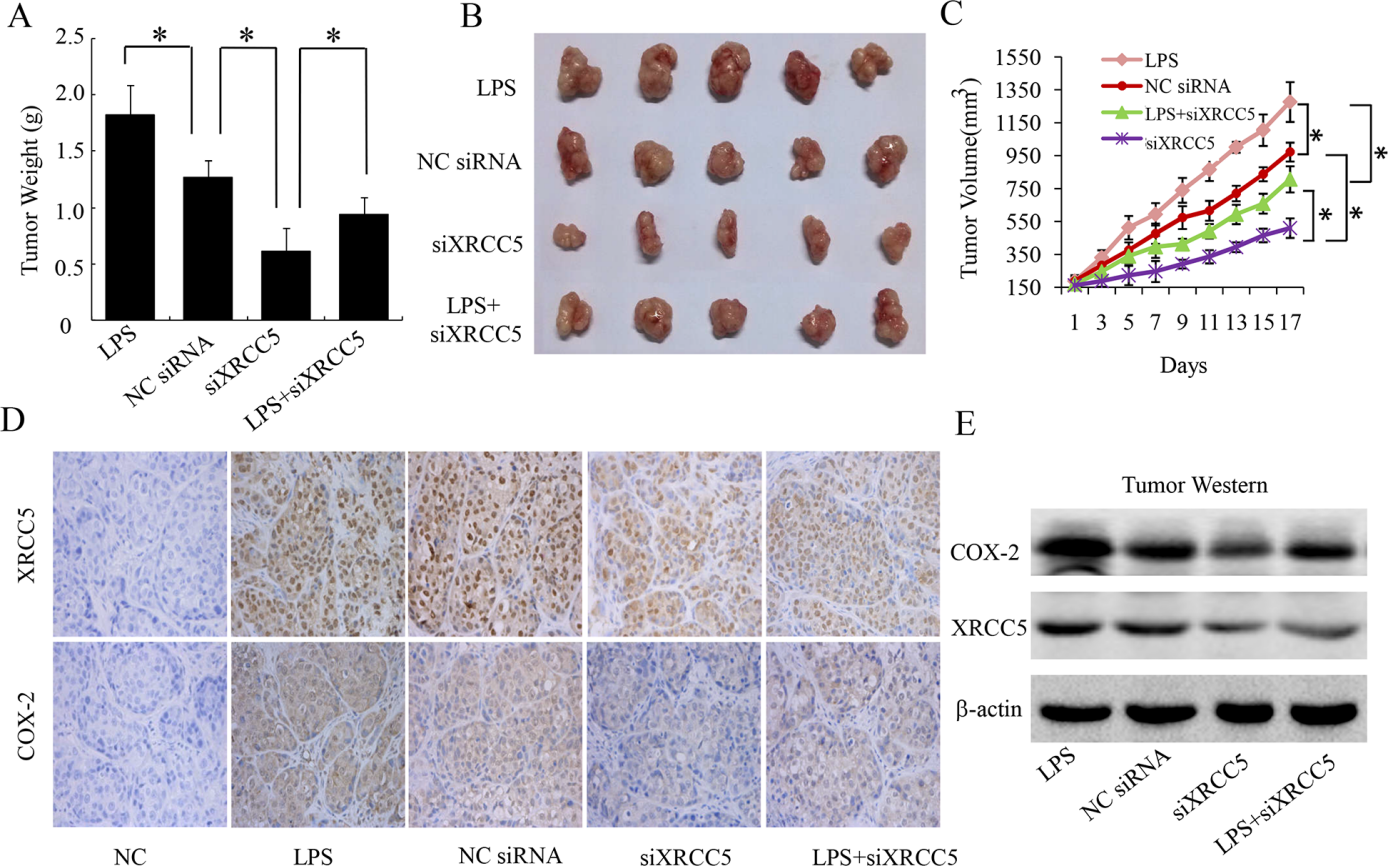
Knockdown of XRCC5 inhibiting tumor growth by down-regulating COX-2 expression in a colon cancer mouse model. (A) Tumor weight at the time of sacrifice of mice in each group. Data are presented as the meane.D. (*P<0.05). (B) Morphology images of each tumor xenograft resected from nude mice seventeen days after first treatment in each group. (C) Tumor growth curves. Tumor volumes at each time point are presented as the mean).D. Tumor growth curves are depicted with tumor volume at each time point. (*P<0.05) (D) Tumor tissue immunohistochemistry of XRCC5 and COX-2. (E) Western blot of XRCC5 and COX-2 with nuclear proteins exacted from tumor tissues. LPS represents lipopolysaccharides, NCsiRNA represents negative control siRNA, and siXRCC5 represents knockdown of XRCC5 with siRNAs.

We further evaluated the effect of XRCC5 on COX-2 expression and tumor growth in nude mice with LoVo cell xenografts. LPS increased tumor weight compared with negative control (NCsiRNA) as shown in Fig 4A. Knockdown of XRCC5 (siXRCC5) decreased tumor weight compared with negative control (NCsiRNA) as shown in Fig 4A. And LPS attenuated the suppression effect of XRCC5 knockdown on tumor weight as shown in Fig 4A. LPS accelerated tumor volume increase compared with negative control (NCsiRNA) as shown in Fig 4B and 4C. Knockdown of XRCC5 (siXRCC5) suppressed tumor growth compared with negative control (NCsiRNA) as shown in Fig 4B and 4C. And LPS attenuated the suppression effect of XRCC5 knockdown on tumor growth as shown in Fig 4B and 4C. Tumor tissue immunohistochemistry showed that LPS increased COX-2 expression and did not affect the expression of XRCC5 as shown in Fig 4D. Tumor tissue immunohistochemistry also showed that siRNA of XRCC5 (siXRCC5) suppressed the expression of XRCC5 successfully as compared with negative control (NCsiRNA), and knockdown of XRCC5 decreased COX-2 expression, as shown in Fig 4D. Moreover, LPS attenuated the suppression effect of XRCC5 knockdown on the expression of COX-2 as shown in Fig 4D. Nuclear proteins of tumor tissues were exacted, XRCC5 and COX-2 were detected with Western blot. Western blot showed that LPS increased COX-2 expression and did not affect the expression of XRCC5as shown in Fig 4E. Western blot also showed that siRNA of XRCC5 (siXRCC5) suppressed the expression of XRCC5 successfully as compared with negative control (NCsiRNA), and knockdown of XRCC5 decreased COX-2 expression, as shown in Fig 4E. Additionally, LPS attenuated the suppression effect of XRCC5 knockdown on the expression of COX-2 as shown in Fig 4E. This *in vivo* experiment in combination with aforementioned *in vitro* cell viability experiment supported that XRCC5 promoted colon cancer growth via up-regulating COX-2.

### XRCC5 interacted with p300 to co-regulate COX-2 expression and promote growth of colon cancer cells

**Fig 5:**
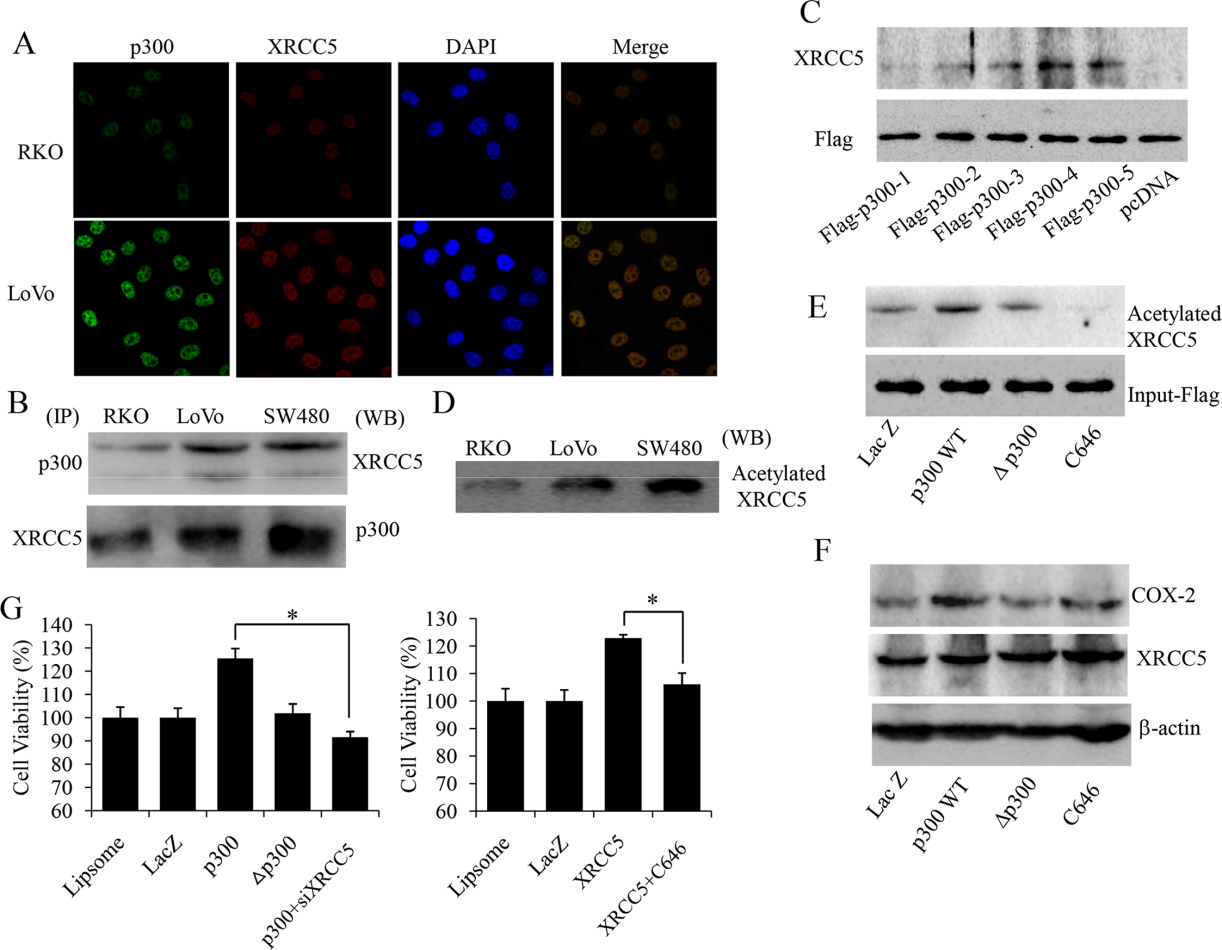
XRCC5 interacting with p300 to co-regulate COX-2 expression in colon cancer cells. (A) Immunofluorescence and confocal microscopy of XRCC5 and p300 in RKO and LoVo cells. XRCC5 is stained by TRITC-conjugated secondary antibodies (red), p300 is stained by FITC-conjugated secondary antibodies (green), and nuclei are stained with DAPI (blue). (B) Co-immunoprecipitation assay of p300 and XRCC5 in RKO, LoVo and SW480 cells.Left: Immunoprecipitation assay (IP) of p300 and XRCC5. Right: Western blot (WB) of XRCC5 and p300. (C) Bottom: The design of the flag-tagged plasmids with different domains of p300. Left: The interaction between XRCC5 and the different domains of p300 detected by immunoprecipitation assay and Western blot. (D)Western blot of XRCC5 with the nuclear extractsimmunoprecipitated by an anti-acetylation antibody in RKO, LoVo and SW480 cells. (E) Western blot of XRCC5 with the nuclear extracts immunoprecipitated by an anti-acetylation antibody in LoVo cells. (F) Western blot of XRCC5 and COX-2 in LoVo cells. (G) MTS cell viability assay in LoVo cells (Left) and RKO cells (Right). Cells treated with liposome negative control is used for data alignment. Data are presented as the meanen.D. (*P<0.05). lacZ represents negative control vector, p300WT represents wild type p300 overexpression, Δp300 represents histone acetyltransferase (HAT) domain deletion mutant p300, C646 represents p300 HAT inhibitor C646, and siXRCC5 represents knockdown of XRCC5 with siRNAs.

p300 (a transcription co-activator) has been reported to participate in the transcription of COX-2 [30]. We further investigated whether p300 cooperated with XRCC5 to regulate COX-2 expression and tumor growth in colon cancer. Immunofluorescence assay revealed that both p300 and XRCC5 proteins were localized in the nucleus, as shown in Fig 5A. Co-immunoprecipitation using nuclear extracts from RKO, LoVo, and SW480 colon cancer cells showed that p300 was present in the immune complexes immunoprecipitated by specific antibodies against XRCC5, and XRCC5 was also present in the immune complexes immunoprecipitated by specific antibodies against p300 vice versa, as shown in Fig 5B. Immunofluorescence assay and co-immunoprecipitation implicated interactions between XRCC5 and p300 in the nucleus of colon cancer cells.

As p300 contained histone acetyltransferase (HAT) domain, we further hypothesized that p300 acetylated XRCC5 to regulate COX-2 expression in colon cancer cells. Western blot showed that overexpression of p300 increased COX-2 expression but not XRCC5 expression in LoVo cells, and p300 HAT inhibitor (C646) decreased COX-2 expression but not XRCC5 expression in LoVo cells, as shown in Fig 5F. However, overexpression of p300 increased XRCC5 acetylation, and p300 HAT inhibitor (C646) decreased XRCC5 acetylation in LoVo cells, as shown in Fig 5E. Acetylated XRCC5 was also constitutively expressed in nuclear proteins of RKO, LoVo and SW480 colon cancer cells, as shown in Fig 5D. All of these indicated that p300 acetylated XRCC5 to regulate COX-2 expression, and acetylated XRCC5 might be the ultimate effecter activating COX-2 promoter.

To further identify the specific domain of p300 for its interaction with XRCC5, we designed p300 overexpression vectors with its five different domains fused to flag tags (data not shown), and overexpressed them in LoVo cells. Nuclear extracts were immunoprecipitated with anti-flag tag antibody, and the precipitated complexes were then blotted with XRCC5 antibody. The results showed that XRCC5 markedly interacted with the domain corresponding to the gene sequence from 1069 to 2414 of p300, as shown in Fig 5C (Flag-p300-4). Notably, this sequence contained the HAT domain of p300. This further supported that p300 acetylated XRCC5 with its HAT domain.

Finally, we evaluated the effect of p300/XRCC5 cooperation on colon cancer cell proliferation. MTS assay indicated that overexpression of p300 increased cell viability, and knockdown of XRCC5 attenuated the promoting effect of p300on cell viability in LoVo cells, as shown in Fig 5G (Left). MTS assay also showed that overexpression of XRCC5 increased cell viability, and p300 HAT inhibitor (C646) attenuated the promoting effect of XRCC5 overexpression on cell viability in RKO cells, as shown in Fig 5G (Right). Collectively, these results demonstrated that p300 interacted with XRCC5 and acetylated the latter to co-regulate COX-2 expression andcell growth of colon cancer cells.

## Discussion

Previous studies indicate that COX-2 promotes carcinogenesis and cancer progression through participating in increasing cell proliferation, inhibiting cell apoptosis, enhancing angiogenesis of tumor, promoting cancer stem cell formation and facilitating immune response shift in tumor microenvironment [11–17]. Additionally, stromal COX-2 can interact with parenchymal COX-2 to promote tumor development and progression [31]. Therefore COX-2 is deemed to be a promising molecular target for cancer treatment. Moreover, clinical data further support COX-2 as a promising target for CRC treatment [18, 19]. However, the preventive and treatment effects of available COX-2 inhibitors (aspirin and celecoxib) on CRC are not ideal, and most CRC patients failed to respond to the available COX-2 inhibitors. Thus elucidating the underlying molecular mechanism of COX-2 participating in CRC development would facilitate novel therapeutic development for CRC.

Gene transcription is rigorously regulated and controlled by the regulatory events occurred at gene promoters and enhancers, transcription factors bind to gene promoters and enhancers to initiate and accelerate gene transcription [32–34]. Promoter region of COX-2 gene includes Sp1 sites, NF-κB sites, AP-2 sites, CAAT enhancer binding protein (C/EBP), TATA-box, Sterol response element (CRE) motif and E-box [35]. Our previous study has shown that XRCC5 binds to the promoter region of COX-2 gene and promotes transcription of COX-2 in lung cancer cells [29]. However, different tumors might have different mechanisms in carcinogenesis and progression. Whether XRCC5 binding to the promoter region of COX-2 gene to promote transcription also exists in CRC needs to be elucidated. To validate this hypothesis, we designed a 479-bpbiotin-labeled double-stranded DNA probe corresponding to the 5’-flanking sequence of the COX-2 gene promoter region to pull down nuclear proteins in four colon cancer cell lines, and mass spectrometry was then used to identify the bound proteins. Results showed that abound protein to COX-2 promoter with a molecular weight of 90-100kDa was identified, and mass spectrometry predicted this protein to be XRCC5. Western blot with a specific antibody against XRCC5 also validated the results from streptavidin-agarose pulldown assay and mass spectrometry analysis. Moreover, luciferase-reporter assay indicated that knockdown of XRCC5 decreased the activity of COX-2 promoter, overexpression of XRCC5 increased the activity of COX-2 promoter, and LPS partially attenuated the suppression effect of XRCC5 knockdown on COX-2 promoter activity. These results support XRCC5 as a transcription factor acting at the promoter region of COX-2 gene to promote COX-2 transcription.

Furthermore, we evaluated the effect of different XRCC5 expressions on colon cancer cell proliferation *in vitro* and growth of tumor xenografts in mice *in vivo*. MTS cell viability assay showed that overexpression of XRCC5 promoted colon cancer cell proliferation, and knockdown of XRCC5 inhibited colon cancer cell proliferation, which suggests that XRCC5 is a proliferation promoting factor in colon cancer cells. Additionally, overexpression of XRCC5attenuated the suppression effect of celecoxib (a COX-2 inhibitor) on colon cancer cell proliferation, which implicates that XRCC5 affects colon cancer cell proliferation via regulating COX-2. Experiments with an animal model with tumor xenografts also indicated that knockdown of XRCC5 inhibited tumor growth, and this suppression effect of XRCC5 knockdownon tumor growth was partially attenuated by LPS. Further tumor tissue immunohistochemistry and Western blot showed that knockdown of XRCC5 decreased XRCC5 expression, and also suppressed COX-2 expression. However, LPS only increased COX-2 expression, but did not affect XRCC5 expression in colon cancer xenografts. These implicate that knockdown of XRCC5 inhibits colon cancer growth *in vivo* via down-regulating COX-2.

XRCC5 is initially recognized to participate in DNA non homologous end joining repair to maintain stability of the whole genome and chromosomes [22]. As instability of genome and chromosomes play key roles in carcinogenesis, XRCC5 is deemed to be an anti-tumor protein [36]. However, the knowledge about the function of XRCC5 in cancer development is changing over time. Previous studies shows that XRCC5 acts as an adherence factor participating in cellular adherence, migration and invasion of tumors [23, 24]. Our early study also shows that XRCC5 promotes proliferation of lung cancer cells via increasing COX-2 transcription [29]. In the present study, we proved that XRCC5 bound to the promoter region of COX-2 gene and increased transcription of COX-2 to promote proliferation of colon cancer cells. So the function of XRCC5 is a double-edged sword, XRCC5 isalso a tumor promoting factor in tumor cells. The exact roles of XRCC5 in preventing normal cell from canceration and participating in the progression of cancer cells need to be evaluated with more studies.

As we know that DNA double strands wrap around histone octamers which are further packaged into condensed chromatin, that hinders interaction between transcription factors with gene promoters and enhancers [37]. CREB-binding protein (CBP) and p300 as transcription co-activator, can acetylate histones with their HAT to relax condensed chromatin [38]. So CBP and p300 might be involved in the mechanism of XRCC5 binding on COX-2 promoter. CBP and p300 proteins share similar structures with four transactivation domains (TADs) [39–44]. TADs of CBP and p300 coordinate and facilitate interactions among basal transcription machinery, general transcription factors and transcription co-activators [39–44]. Additionally, HAT of CBP and p300 not only acetylates histones to relax condensed chromatin, but also acetylates transcription factors to regulate their binding ability to promoters and enhancers. Our previous study showed that CBP acetylated XRCC5 to regulate its ability to bind to the promoter region of COX-2 gene in lung cancer cells [29]. In the present study, immunofluorescence assay revealed that both p300 and XRCC5 proteins were co-localized in nuclei of colon cancer cells, co-immunoprecipitation experiment also proved interaction between p300 and XRCC5 in colon cancer cells. Further immunoprecipitation experiment identified the specific domain of p300 for its interaction with XRCC5 to be the HAT domain. Western blot also showed that in colon cancer cells, overexpression of p300 increased COX-2 expression and acetylation of XRCC5, HAT inhibitor (C646) of p300 decreased COX-2 expression and acetylation of XRCC5. However, overexpression of p300 and its HAT inhibitor (C646) did not affect total expression of XRCC5 in colon cancer cells. So we deduces that acelytated XRCC5 is the ultimate effecter protein binding to the region of COX-2 promoter. Cell viability assay showed that overexpression of p300 increased cell viability, knockdown of XRCC5 attenuated the promoting effect of p300 on cell viability. This implies that overexpression of p300 increased cell viability via acetylating XRCC5. Cell viability assay also showed thatp300 HAT inhibitor (C646) attenuated the promoting effect of XRCC5 on colon cancer cell viability, which implicates that acetylation by p300 is essential for XRCC5 to promote colon cancer cell proliferation. These observations suggest that p300 acetylates XRCC5 to up-regulate COX-2 expression to promote colon cancer growth. However, we also found that COX-2 expression was not completely dependent on XRCC5 expression, which implies multiple signaling pathways involved in the regulation of COX-2 expression. Further studies are needed to elucidated the mechanisms of interaction of multiple signaling pathways in the regulation of COX-2.

## Conclusion

In summary, our current study has revealed that XRCC5 acts as a transcription factor binding to the promoter region of COX-2 and up-regulating the activity of COX-2 promoter in colon cancers. p300 interacts with XRCC5 by its HAT acetylating XRCC5 in colon cancers. p300 cooperates with XRCC5 to regulate COX-2 expression through acetylating XRCC5 to promote colon cancer growth *in vitro* and *in vivo*. Targeting the XRCC5/p300/COX-2 signaling pathway is a potentially promising strategy in the treatment of colon cancers.
